# A hairy tail in the stomach: A silent warning

**DOI:** 10.12669/pjms.41.6.11387

**Published:** 2025-06

**Authors:** Nazish Butt, Lajpat Rai, Ghulam Mohiuddin

**Affiliations:** 1 Nazish Butt Associate Professor, Head of Gastroenterology Department, Jinnah Postgraduate Medical Center, Karachi, Pakistan; 2 Lajpat Rai Consultant Gastroenterologist, Jinnah Postgraduate Medical Center, Karachi, Pakistan; 3 Ghulam Mohiuddin Resident Gastroenterology (PGY-5), Jinnah Postgraduate Medical Center, Karachi, Pakistan

**Keywords:** Trichotillomania, Trichobezoars, Rapunzel syndrome, Trichophagia

## Abstract

Trichotillomania is a psychiatric disorder in which one is urged to pull hairs and eyelashes out and one is urged to eat these pulled-out hairs, resulting in Rapunzel syndrome (a rare disorder where gastric trichobezoars traverse into the small intestine and can lead to intestinal obstruction). Here we present two case reports. In the First case, a 17 years old boy presented with a history of recurrent pancreatitis and microcytic anemia, while the other patient was a 16 years old female presenting with complaints of epigastric pain and non-projectile, foul-smelling vomiting associated with a low-grade fever. Both had a history of depression and compulsive hair-pulling and eating. After successful surgical management, both patients were eventually referred to Psychiatry.

## INTRODUCTION

A bezoar is a mass of indigestible material in the gastrointestinal (GI) tract.^1^ Based on the difference in composition, they are classified as Trichobezoar, Phytobezoar, Pharmacobezoar, and Lactobezoar. Phytobezoar is the most common variety and is composed of indigestible plant material such as fruits, fibers, skin, and seeds. It is associated with reduced gastric acidity, delayed motility, a history of gastric surgery, and poor gastric mixing.^2^ Patients having this condition usually remain asymptomatic for years until the bezoar increases in size. In comparison, others present with upper GI bleeding, ulceration, anorexia, weight loss, obstructive jaundice, acute pancreatitis, and gastric emphysema.^3^ These can also present with malabsorption complicated by iron deficiency, protein-losing enteropathy, and megaloblastic anemia. They can also result in an atypical gastrointestinal syndrome referred to as Rapunzel syndrome, an infrequent disorder due to gastric trichobezoars migration to the small intestine, leading to complications.^4^

## CASE PRESENTATION

### Case-01:

**Fig.1:**
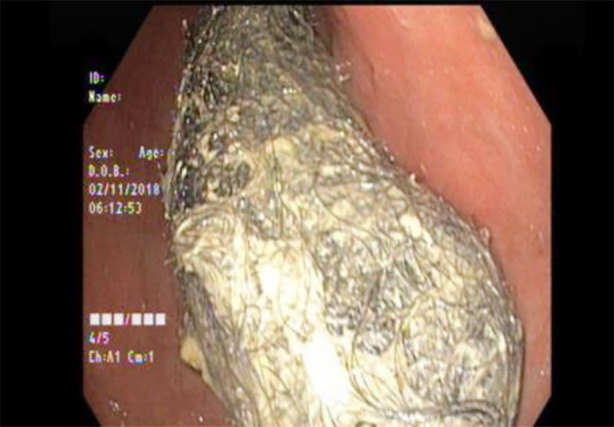
EGD showed a large trichobezoar in the stomach passing through the pylorus.

**Fig.2:**
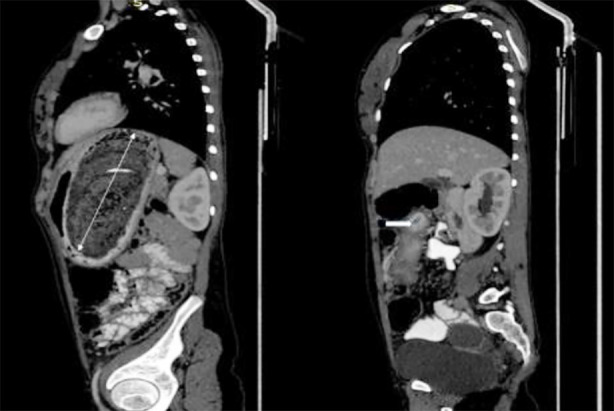
CT scan abdomen with contrast sagittal view, more prominent D2: showing a large heterogeneous intraluminal mass with a mottled gas pattern in the stomach, which extends through the pylorus up to the second part of the duodenum.

A 17 years old boy with a history of Psychiatric illness was admitted with a history of recurrent attacks of pancreatitis. There was no history of alcohol intake or substance abuse. On physical examination, he was hemodynamically stable and pale. Abdominal examination revealed epigastric tenderness and guarding. Blood tests showed: Hemoglobin of 5.9 g/dL, MCV 54.7 FL, Platelets 726000, Leucocytes 9200, Serum lipase 360 U/L, Serum amylase 145 U/L, serum albumin 2.28 g/dL, INR 1.92, and Lactate Dehydrogenase 486 U/L. His renal profile, electrolytes, liver function tests, IgG4 levels, serum calcium, and triglyceride levels were in normal ranges. EGD revealed a large trichobezoar in the stomach extending beyond the pylorus reaching up to the duodenum causing Gastric outlet obstruction (GOO) and ampullary obstruction (Fig.1). CT scan of the abdomen revealed a large gastric bezoar and acute pancreatitis with moderate ascites (Fig.2). Endoscopic retrieval of trichobezoar was attempted with a snare but it failed because of its large size.

### Case-02:

**Fig.3:**
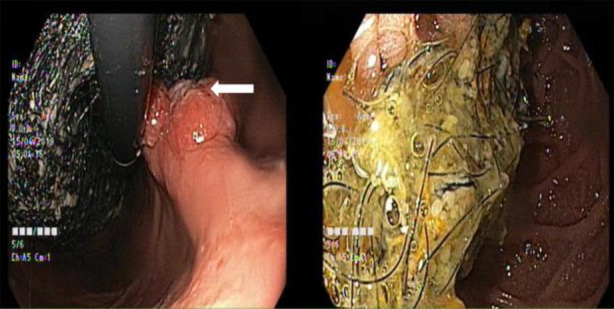
Endoscopic view showed a large trichobezoar in the stomach starting from GEJ and extending up to D2, causing luminal narrowing with a large (FOREST CLASS III) ulcer and a sessile mass in the gastric body (white arrow).

A 16 years old girl with a known case of depression presented with complaints of abdominal pain for three months and foul-smelling vomiting for one month. There was a history of depression for five years and compulsive hair-pulling and eating for six to eight months. On abdominal examination, there was a mass in the epigastric region extending to the right hypochondriac region; it was approximately 5 x 6 cm, smooth, non-tender, with round margins, firm in consistency, and not freely movable. EGD showed a large trichobezoar extending up to the duodenum and flat gastric lesions (Fig.3).

**Fig.4:**
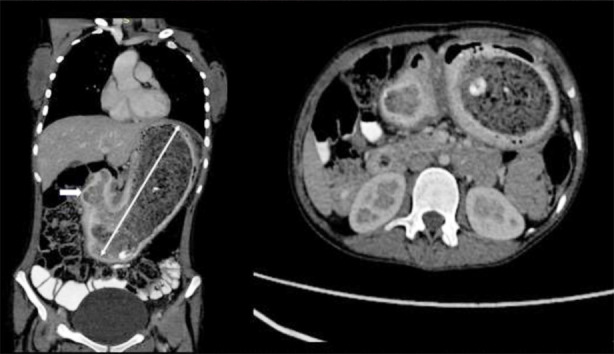
CT scan of the abdomen with contrast, coronal and axial sections: showing a large mass of heterogeneous material in the stomach, extending through the pylorus into the duodenum suggestive of a large trichobezoar (Rapunzel Syndrome).

Her X-ray abdomen was done, which showed a radiolucent area in the stomach. CT scan abdomen revealed findings suggestive of gastric bezoar (Fig.4). Histopathology revealed Helicobacter Pylori gastritis. The surgical team was involved in both patients’ treatment. After successful surgical removal, the patient was ultimately referred for Psychiatric management.

## DISCUSSION

Rapunzel syndrome is an unusual entity with nothing more than 40 cases reported.^3^ It was discovered by Vaughan in 1968^4^, was first reported in the literature by Baudomant in 1779, and the first surgical removal was performed in 1883 by Schonbern.^5^

We encountered two patients, one a boy of 17 years old, who has a psychiatric illness and was having complaints of recurrent Pancreatitis, the other was a young female of 16 years old who also has a psychiatric history and she has also the history of pulling her hair and eating for 6-8 months. To compare our study with national studies, we observe that most patients in other studies were female and presented with similar symptoms, such as acute abdomen.^6^ The mainstay of management was surgical intervention.^7^ In contrast, when we look at international studies, we find that patients often benefit from a more holistic approach, including psychiatric evaluation and long-term management and follow-up.

Our study highlights the importance of early psychiatric evaluation for common psychiatric disorders, as this can help prevent serious complications associated with Rapunzel Syndrome. Patients often present at later stages of the disease due to a lack of proper investigations for any underlying conditions. By addressing the issue at its early stages, we can potentially prevent the progression of this syndrome.

It is more common in females with psychiatric disorders and learning disabilities who have the habit of pulling out their hair (trichotillomania) and then swallowing it (Trichophagia).^8^ Other associations include psychological disturbances like abuse, pica, depression, obsessive-compulsive disorder, and anorexia nervosa.^9^ The usual site for trichobezoar is the stomach, but they may extend up to the duodenum, jejunum, ileum, and even the large intestine: a condition called Rapunzel syndrome.^10^

Human hairs resist digestion, and peristalsis does not affect their smooth surface. This leads to its accumulation in the mucosal folds of the stomach. With time, repeated ingestion of hair causes impaction together with the mucus and food particles, forming a trichobezoar. Hairballs become more compact with time, and gastric churning traps further new hair, mucus gives it a shiny surface, and proteins denaturing in the stomach environment give it a black color. The foul smell of this trichobezoar results from the fermentation of fats.^10^ The hair is usually from the scalp, but the source can be the eyelashes, eyebrows, and pubis. Rarely do patients with this disorder chew from artificial sources like those of wigs.^11^

Trichobezoar can be detected incidentally because of a lack of awareness by the guardians. Although uncommon, these can lead to devastating complications, including death if left untreated. It can be diagnosed easily by several modalities, including abdominal ultrasonography and/or CT scan. However, EGD is a more effective diagnostic tool. Moreover, EGD helps to differentiate a trichobezoar from another foreign body. The definitive treatment is surgical removal followed by psychiatric and behavioral therapy to prevent a recurrence.^12^ Several options can be considered for the removal of trichobezoar, the treatment of choice being open surgery (laparotomy), due to its extent depending on the size and site of trichobezoar.^12^ Other options are laparoscopic or endoscopic removal, or the use of chemicals. In recurrent and resistant cases, treatment of the underlying cause i.e. Cognitive Behavioral Therapy (CBT) and pharmacology, is indicated.

## CONCLUSION

Physicians should think of trichobezoar as a differential diagnosis in young adults presenting with abdominal pain/mass or GOO having a history of psychiatric disorders. Surgical or endoscopic removal of this mass is the treatment of choice. Psychiatric evaluation and behavioral therapy are mandatory to prevent a recurrence.

### Authors’ contribution:

**NB:** Who did the endoscopy and supervised the editing process, made the necessary changes.

**GM and LR:** Went through the literature review and wrote the case report.

**NB:** Is responsible and accountable for the accuracy and or integrity of the work.
